# Supplementary material for: Adult picky eating and associations with childhood picky eating, maternal feeding, aversive sensory responsiveness, disgust and obsessive-compulsive symptoms
Source: PeerJ. 2025 May 16;13:e19444. doi: 10.7717/peerj.19444 (PMC12087578; doi:10.7717/peerj.19444)
Supplement: Supplemental Information 2 [file peerj-13-19444-s002.docx]

Age= years of age

Hight=height in cm

Weight=weight in kg

Education=years of education

Sex= male/female/other

New Immigrant=1=yes/2=no

Year of immigration=just that

Language=mother tongue: 1=Hebrew/2=English/3=Arabic/4=Russian/5-Spanish/6=Other

Religious tendency: 1=religious/2=secular/3=traditional/4=ultraorthodox/5=other

Religion:1=Jewiah/2=Moslem/3=Christian/4=Druze/5=areligious-atheist/6=other

Family status:1=married/2=divorced/3=single/4=cohabiting/5=widowed/6=other

Nutrition: “How would you describe your main nutrition?” 1=strictly vegetarian/2=vegetarian and pescatarian/3=vegan/4=observe Jewish dietary law/5-unlimited nutrition/6=avoid milk and diary products/7=other

Reason for nutrition: “if you have some form of limitation on your diet – what is the reason?” 1=Jewish diwtary law/2=medical reason/3=ideaology/4=personal preferences/5=nutrition is not limited

Developmental delay: “to the best of your knowledge, did you suffer from a developmental delay?”1=yes/2=no/3=don’t know

Eating problems:”To the best of your knowledge, did you suffer from an eating problem in childhood?” 1=yes/2=no/3=dpn’t remember

Medical problems: “Did you suffer from medical problems in your childhood?” 1=ADHD/2=digestive problems/3=sense problems such as wision or hearing/4=internal problems such as asthma or respiratory disease/5=emotionalor social problems/6=problems arising from birth complications such as CP/7=other/8=I did not have any medical problems in childhood/9=I don’t know

Medical diagnosis: In your childhood, were you diagnosed with a medical condition”? 1/=yes/2=no

Medical_diagnosis_known – free field description

Medical diagnosis suffering : 1=I had medical problems in childhood and I am still contending with them/2=I am no longer experiencing any difficulty from the medical problems I contended with in childhood/3=I had no medical problems in childhood nor do I have any currently/4=I had no medical problems in childhood but I have some currently

Suffering scale: “If you are currently contending with a medical condition, to what extent is it affecting you?” Responses on a sliding scale 1 = not at all to 100 = completely debelitating

The follwing questionnaires were used in this study:

Adult Picky Eating Questionnaire [APEQ], obsessive-compulsive inventory revised [OCI-R], sensory processing questionnaire [SRQ-IS], general disgust propensity [DPSS-12], food disgust [FDQ-S], and three maternal feeding practices [RCFQ].

APEQ response categories are on a five-point Likert scale ranging from 1 “Never” to 5 “Always.”

OCI-R response categories are on a five-point Likert scale ranging from 1 “Not at all” to 5 “Extremely”

SRQ-IS response categories are on a five-point Likert scale of intensity ranging from 1 “Not at all” to 5 “Very much” with an additional option of 0 “I have never experienced”

DPSS-12 response categories are on a five-point Likert scale ranging from 1 “Never” to 5 “Always”

FDQ-S response categories are on a five-point Likert scale ranging from 1 “No feeling of disgust” to 5 “Strong feeling of disgust”

RCFQ - response categories are on a five-point Likert scale ranging from 1 “Do not agree at all” to 5 “Agree.”
