# Supplementary material for: Adult picky eating and associations with childhood picky eating, maternal feeding, aversive sensory responsiveness, disgust and obsessive-compulsive symptoms
Source: PeerJ. 2025 May 16;13:e19444. doi: 10.7717/peerj.19444 (PMC12087578; doi:10.7717/peerj.19444)
Supplement: Supplemental Information 4 [file peerj-13-19444-s004.docx]

The follwing questionnaires were used in this study:

Adult Picky Eating Questionnaire [APEQ], obsessive-compulsive inventory revised [OCI-R], sensory processing questionnaire [SRQ-IS], general disgust propensity [DPSS-12], food disgust [FDQ-S], and three maternal feeding practices [RCFQ].

APEQ response categories are on a five-point Likert scale ranging from 1 “Never” to 5 “Always.”

OCI-R response categories are on a five-point Likert scale ranging from 1 “Not at all” to 5 “Extremely”

SRQ-IS response categories are on a five-point Likert scale of intensity ranging from 1 “Not at all” to 5 “Very much” with an additional option of 0 “I have never experienced”

DPSS-12 response categories are on a five-point Likert scale ranging from 1 “Never” to 5 “Always”

FDQ-S response categories are on a five-point Likert scale ranging from 1 “No feeling of disgust” to 5 “Strong feeling of disgust”

RCFQ - response categories are on a five-point Likert scale ranging from 1 “Do not agree at all” to 5 “Agree.”
